# A Novel Circulating miRNA-Based Model Predicts the Response to Tripterysium Glycosides Tablets: Moving Toward Model-Based Precision Medicine in Rheumatoid Arthritis

**DOI:** 10.3389/fphar.2018.00378

**Published:** 2018-05-24

**Authors:** Yanqiong Zhang, Hailong Wang, Xia Mao, Qiuyan Guo, Weijie Li, Xiaoyue Wang, Guangyao Li, Quan Jiang, Na Lin

**Affiliations:** ^1^Institute of Chinese Materia Medica, China Academy of Chinese Medical Sciences, Beijing, China; ^2^Division of Rheumatology, Guang An Men Hospital, China Academy of Chinese Medical Science, Beijing, China; ^3^Department of Rheumatology, Basic Medical College of Guiyang University of Chinese Medicine, Guiyang, China

**Keywords:** precision medicine, rheumatoid arthritis, *Tripterygium wilfordii* Hook F-based therapy, microRNA biomarker, molecular interaction network

## Abstract

Accumulating clinical evidence show that not all rheumatoid arthritis (RA) patients benefit to the same extent from a *Tripterygium wilfordii* Hook F (TwHF)-based therapy-Tripterysium glycosides tablets (TG tablets), which emphasizes the need of predictive biomarkers and tools for drug response. Herein, we integrated TG tablets' response-related miRNA and mRNA expression profiles obtained from the clinical cohort-based microarray, miRNA target prediction, miRNA-target gene coexpression, as well as gene-gene interactions, to identify four candidate circulating miRNA biomarkers that were predictive of response to TG tablets. Moreover, we applied the support vector machines (SVM) algorithm to construct the prediction model for the treatment outcome of TG tablets based on the levels of the candidate miRNA biomarkers, and also confirmed its good performance via both 5-fold cross-validation and the independent clinical cohort validations. Collectively, this circulating miRNA-based biomarker model may assist in screening the responsive RA patients to TG tablets and thus potentially benefit individualized therapy of RA in a daily clinical setting.

## Introduction

Rheumatoid arthritis (RA) represents a systemic autoimmune disorder that is characterized by chronic arthritis, synovium hyperplasia, bone and cartilage erosion, as well as joint swelling and destruction. *Tripterygium wilfordii Hook F* (TwHF), a traditional Chinese herb, has an obvious effect on relieving joint pain. TwHF-based therapy has been extensively used in the treatment of RA as a disease-modifying anti-rheumatic drug (DMARD) for many years in China (Bao and Dai, 2011; Wang et al., 2016). Tripterysium glycosides tablets (TG tablets, Leigongteng Duogan tablets), which are extracted from TwHF, are marketed as herbal medications for RA patients with better therapeutic effects than several first-line DMARDs according to multiple randomized controlled trials (Chang et al., 1999; Lv et al., 2015; Wang et al., 2016, 2017). For example, Wang et al. (2016) reported that TG tablets alone attained a markedly higher modified American College of Rheumatology Criterion of 20% (ACR 20) response, compared with methotrexate, leflunomide, sulphasalazine, tacrolimus, minocycline; Lv et al. (2015) observed that the combination of methotrexate and TG tablets was better than methotrexate monotherapy in controlling disease activity in patients with active RA. However, several limitations have found to be existed in the treatment of TG tablets, including the reproductive system damage, gastrointestinal discomfort, amenorrhea, and individualized differences (Xi et al., 2017). Notably, about 30% of RA patients treated with TG tablets fail to achieve clinical improvement, which emphasizes the need of predictive biomarkers and tools.

The aberrant changes in the expression and/or function of microRNAs (miRNAs) may result in many pathological conditions, such as cancers, infection, and autoimmune diseases (Eulalio and Mano, 2015). Since the existence with an extremely high stability in the serum and plasma, miRNAs derived from peripheral blood and body fluid have been regarded as promising biomarkers for diagnosis and prognosis of certain diseases (Gibbings et al., 2009). In recent years, an increasing number of miRNAs have been found to be dysregulated in the different stages of RA progression (Pauley et al., 2008; Zhu et al., 2012; Dong et al., 2014; Castro-Villegas et al., 2015; Mallinson et al., 2017). For example, Castro-Villegas et al. (2015) identified a specific plasma miRNA signature (miR-23 and miR-223) that may serve both as predictor and biomarker of response to anti-TNFα/DMARDs combination therapy; Mallinson et al. (2017) confirmed three miRNAs, miR-26b-5p, miR-487b-3p, and miR-495-3p, as stratification biomarkers for response to allogeneic adipose-derived mesenchymal stem cells in RA. These findings suggesting that miRNAs may be used to help monitor disease severity and therapy outcome.

**Figure 1:**
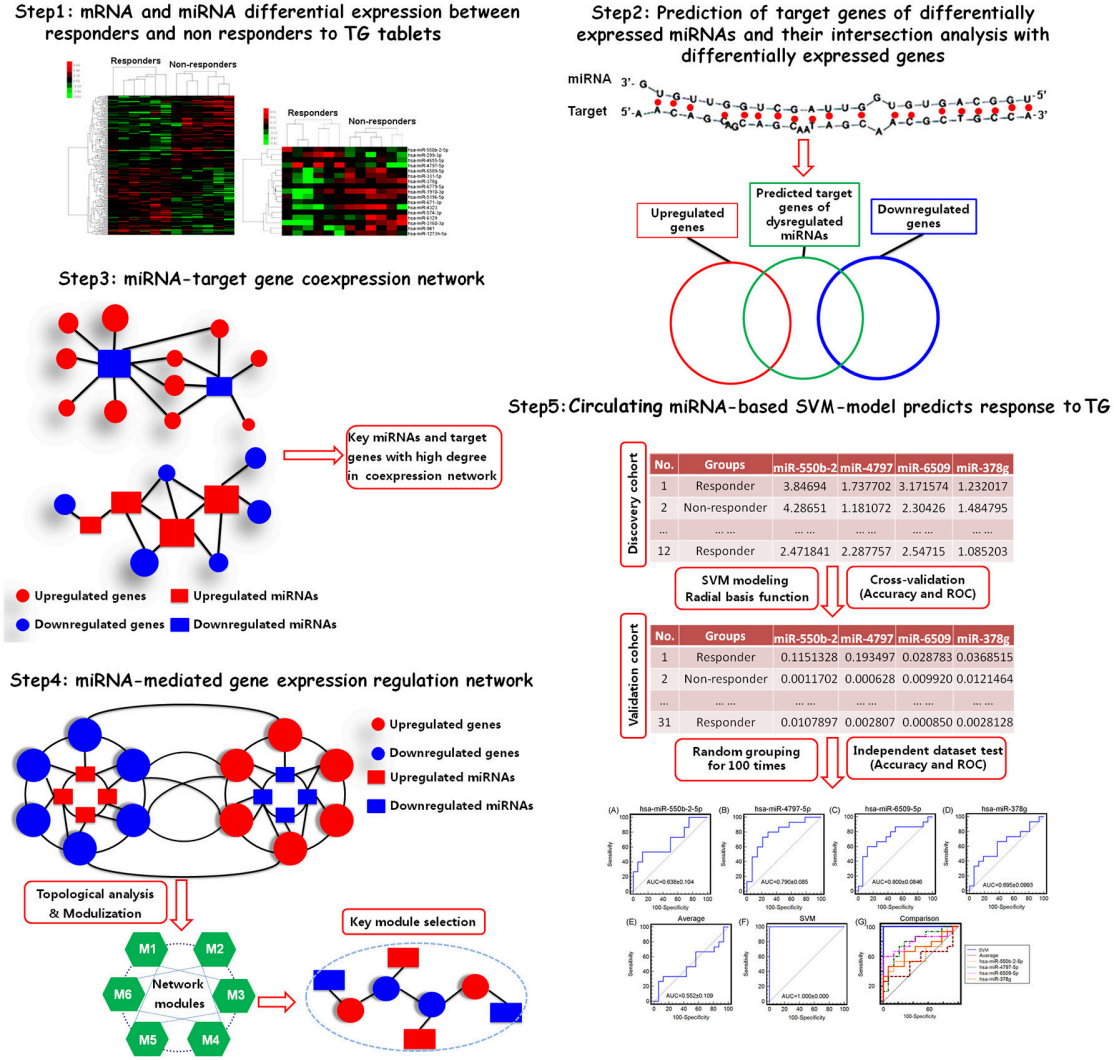
A schematic diagram of the systematic strategies to identify circulating miRNA biomarkers and to construct SVM-based model that predictive of response to TG tablets.

Yet, to date no study has been performed to evaluate the differences in expression profile of circulating miRNAs between responsive and non-responsive RA patients to TwHF-based therapy. In the current study, we detected miRNA and mRNA expression profiles in peripheral blood mononuclear cells (PBMCs) obtained from a discovery cohort including 12 RA patients (6 responders and 6 non-responders) treated with TG tablets by Affymetrix miRNA 4.0 and EG1.0 arrays, respectively. Then, a list of candidate miRNA biomarkers associated with response to TG tablets were identified by integrating differential expression data analysis, miRNA target gene prediction, miRNA-target gene coexpression network and miRNA-mediated gene signal transduction network analyses. After that, a support-vector-machine (SVM) model based on the levels of the candidate miRNA biomarkers in RA patients was constructed and its predictive performance was also evaluated by 5-fold cross-validation test and independent dataset test based on a validation cohort including 31 RA patients (15 responders and 16 non-responders). The technical strategy of this study was illustrated as Figure 1.

## Materials and methods

### Ethics statement

This study was performed according to the guidelines of the Declaration of Helsinki for humans and was approved by the Research Ethics Committee of Guang'anmen Hospital. The informed and written consent were obtained from all patients.

### Patients

A total of 43 RA patients (10 men and 33 women, aged from 25 to 84 years old, median age = 57.3 years old) were enrolled from January 2015 through June 2017 in Division of Rheumatology, Guang'anmen Hospital. Inclusion Criteria included (1) A diagnosis of active RA based on the American College of Rheumatology (ACR) 1987 criteria for RA or the 2010 ACR/ European League against Rheumatism (EULAR) Criteria (Aletaha et al., 2010); (2) A symptom duration of <1 year; (3) No use of DMARDs previously; (4) Availability of clinical and laboratory parameters at initiation of TG tablets and after 3 months and availability of serum samples. Patients received oral TG tablets (20 mg.tid.po, purchased from Zhejiang Deengde Co., Ltd. Z33020422, Xinchang, Zhejiang) for 12 weeks. Responders to TG tablets were defined as patients who were treated with TG tablets for 12 weeks achieved ACR 20, and non-responders were defined as patients who were treated with TG tablets for 12 weeks but not achieved ACR 20 (American College of Rheumatology Subcommittee on Rheumatoid Arthritis, 2002).

All 43 RA patients were divided into two cohorts: a discovery cohort (*n* = 12, 6 responders and 6 non-responders) and a validation cohort (*n* = 31, 15 responders and 16 non-responders). The former was used to detect the miRNA and mRNA expression profiles in peripheral blood mononuclear cells (PBMCs) and to train SVM model predictive of response to TG tablets; The latter was used to validate the levels of candidate miRNA biomarkers of response to TG tablets by qPCR assay and to evaluate the predictive efficiency of SVM-based model. Clinical and laboratory parameters of the two cohorts including the treatment protocols were shown in Table 1 and Table S1.

**Table 1:** Clinical and laboratory parameters of RA patients enrolled in the current study.

**Parameters**	**Discovery cohort (*n* = 12)**	**Validation cohort (*n* = 31)**
Age (years, mean, range)	55.9 (45–80)	57.9 (25–84)
Gender (male/female)	2/10	8/23
Erythrocyte sedimentation rate (ESR, mm, mean)	58.4	40.5
C-reactive protein (CRP, mg/dL, mean)	25.6	21.5
Positive rheumatoid factor (*n*, %)	10,83.3	24,77.4
Positive anti-cyclic citrullinated peptide (CCP) antibodies (*n*, %)	9,75	24,77.4

### mRNA and miRNA expression profiling

mRNA and miRNA expression profiles in PBMCs obtained from responders and non-responders of TG tablets were respectively detected using Affymetrix miRNA 4.0 and EG1.0 arrays carried out by Shanghai GMINIX Biotechnology Corporation, Shanghai, China. The miRNA and mRNA expression microarray data of GSE106894 and GSE106893 were respectively obtained from the National Center of Biotechnology Information Gene Expression Omnibus.

### Screening of differentially expressed miRNAs and mRNAs

Lists of miRNAs and mRNAs with significantly differential expression between responder and non-responder groups were identified using the criteria of |log2 fold change (FC)| > 0.5 and *P* < 0.05 by the RVM *t*-test, which can raise degrees of freedom effectively in the cases of small samples. After the significant analysis and the False Discovery Rate (FDR) analysis, we selected the differentially expressed genes according to the *p* < 0.05 and FDR (≥1.0) threshold. The heat map package in R (version 1.0.2, R Core Team, Vienna, Austria) was used for the hierarchical clustering analysis. The basic idea of the hierarchical clustering analysis is to assemble a set of genes into a tree, where genes are joined by very short branches if their expression patterns are very similar to each other, and by increasingly longer branches as their similarity decreases. The first step in hierarchical clustering is to calculate the distance matrix between the gene expression data. Once this matrix of distances is computed, the clustering begins. Agglomerative hierarchical processing consists of repeated cycles where the two closest remaining genes (those with the smallest distance) are joined by a node/branch of a tree, with the length of the branch set to the distance between the joined genes. The two joined genes are removed from list of genes being processed and replaced by a gene that represents the new branch. The distances between this new gene and all other remaining genes are computed, and the process is repeated until only one gene remains.

### miRNA target prediction

Candidate target genes of differentially expressed miRNAs were predicted by two different online programs, including TargetScan (Release 7.1, http://www.targetscan.org/) (Lewis et al., 2005) and miRanda (Last Update: 2010-11-01, http://www.microrna.org/microrna/home.do) (John et al., 2004). The common prediction results obtained from TargetScan and miRanda were retained to ensure high accuracy.

### miRNA-target gene co-expression network analysis

To build a miRNA-target gene co-expression network, the relationship between miRNAs and the corresponding putative target genes was counted by their levels in responders and non-responders of TG tablets. The center of the network was represented by degree, referring to the contribution of one miRNA to the target genes around or the contribution of one target gene to the miRNAs around. The key miRNA in the network always have the biggest degree.

### miRNA-mediated gene signal transduction network analysis

miRNA-mediated gene signal transduction network was constructed using interactions among differentially expressed genes which were also the putative targets of differentially expressed miRNAs between responder and non-responder groups. The considered evidence of gene-gene interactions was obtained from the public database STRING (Search Tool for Known and Predicted Protein-Protein Interactions, version 10.0, http://string-db.org/). Interactions with a combined score higher than the median value of all of the combined scores were selected. In the network, nodes represented differentially expressed genes which were also the putative targets of differentially expression miRNAs, and edges represented interactions between the nodes.

To identify the most important nodes in the network, four topological features were calculated based on the following definition: (1) Node's degree: the sum of connection strengths of node i with the other genes, which measures how correlated a gene is with all other genes in a network; (2) Node's betweenness measures reflecting the importance of a node in a network relative to other nodes; For a graph G:(V,E) with n vertices, the relative betweenness centrality ${C^{\prime }}_{B}\left(v\right)$ is defined by: ${C^{\prime }}_{B}\left(v\right)=\frac{2}{n^{2}-3n+2}\underset{\begin{array}{c} s\neq v\neq t\in V\\ s\neq t \end{array}}{\sum }\frac{\sigma_{st}\left(v\right)}{\sigma_{st}}$ where σ_*st*_ is the number of shortest paths from s to t, and σ_*st*_(*v*) is the number of shortest paths from s to t that pass through a vertex v. (3) Node's closeness measures how long it will take to spread information from node i to all other nodes sequentially; Closeness *C*_(*i*)_ is defined as the inverse of the farness which is the sum of node i distances to all other nodes and calculated by $C_{\left(i\right)}=\frac{1}{\sum_{y}d\left(y,i\right)}$, where *d*(*y, i*) is the distance between vertices *i* and*y*; The larger a node's degree/node betweenness/ closeness is, the more important the node is in the network; (4) Network modularization: genes that are highly interconnected within the network are usually involved in the same biological modules or pathways; The Markov clustering algorithm was used to divide all nodes into different functional modules (Jansen et al., 2002; Spirin and Mirny, 2003; Wei and Li, 2007; Li and Li, 2008; Zhang and Wiemann, 2009).

“Edge Betweenness” was calculated to assess the importance of a specific interaction in the network. It is defined as the frequency of an edge that places on the shortest paths between all pairs of vertices in network (Narayanan et al., 2011). The gene-gene interaction with the highest edge-betweenness value is most important one during the signal transduction among nodes in the network.

### Construction of circulating miRNA-based SVM-model and evaluation of model performance by 5-fold cross-validation

SVM algorithm, in which a radial basis function [$K\left(x_{i},x_{j}\right)=\exp\left(-\gamma {\left||x_{i}-x_{j}|\right|}^{2}\right),\gamma >0$] was chosen as the kernel function, was used to construct our circulating miRNA-based model that was predictive of response to TG tablets, and cross-validation method was utilized to evaluate the performance of the model. The software LIBSVM 3.20 was employed (Practical guide to support vector classification. http://www.csie.ntu.edu.tw/) and the levels of 4 candidate miRNA biomarkers in the discovery cohort were used as inputs. For a given test case*i*, the SVM-model outputs a predictive result as*j*, where*j* ∈ (+1, −1). This predictive result refers to the distance of case *i* from the optimal separating hyperplane in the feature space, and indicates the class *j* to which case *i* belongs.

For 5-fold cross-validation, the levels of 4 candidate miRNA biomarkers in the discovery cohort was divided into two parts: training dataset and testing dataset. Due to the small sample size of the discovery cohort, the 5-fold cross-validation (five times) was performed. The average *Accuracy*, *Sensitivity* and *Specificity*, as well as the average area-under-curve (*AUC*) from receiver-operating-characteristic (ROC) curves were calculated as the following formula:

(f1) $$\begin{array}{r} Sensitivity=\frac{TP}{TP+FN} \end{array}$$

(f2) $$\begin{array}{r} Specificity=\frac{TN}{TN+FP} \end{array}$$

(f3) $$Accuracy=\frac{\sum TP+TN}{N}$$

where *TP*, *TN*, *FP*, *FN* respectively refer to the number of true positive, true negative, false positive and false negative result components in a test, while *N* refers to the total number of predicted samples.

### Quantitative PCR analysis

To evaluate the predictive performance of our circulating miRNA-based SVM-model using independent dataset test, quantitative PCR analysis for miRNAs in the model was performed using the peripheral blood samples obtained from the validation cohort according to our previous studies (Lin et al., 2014; Zhang et al., 2014). U6 small RNA was respectively used as internal controls for miRNA expression normalization and quantification. Quantitative PCR analysis and data collection were performed on the ABI 7900HT qPCR system using the primer pairs listed in Table S2. Relative quantification of miRNA expression was evaluated using the comparative cycle threshold (CT) method. The raw quantifications were normalized to U6 values for each sample and fold changes were shown as mean ± SD in three independent experiments with each triplicate.

### Evaluation of model performance by independent dataset test

The differential expression patterns of candidate miRNA biomarkers and the performance of our circulating miRNA-based SVM-model that were predictive of response to TwHF were both validated by the independent dataset test using the validation cohort. The average accuracy and area under ROC curves (AUC) were calculated as formula *f1*~*3*.

### Statistical analyses

Statistical analyses were performed using SPSS software (Version 13.0, Statistical Program for Social Sciences, Inc:Chicago, IL, USA). miRNA and mRNA levels between the responder and non-responder groups were compared by one-way analysis of variance. *P* < 0.05 were considered significant.

## Results

### Differentially expressed mRNAs and miRNAs associated with response to TG tablets

The differences in patients' response to TG tablets were assessed by comparing miRNA and mRNA expression profiles in PBMC between responder and non-responder groups. A total of 17 differentially expressed miRNAs (4 upregulated and 13 downregulated) and 212 differentially expressed genes (102 upregulated and 110 downregulated) were identified (all fold change > 1.67 and *P* < 0.05, Table S3).

**Figure 2:**
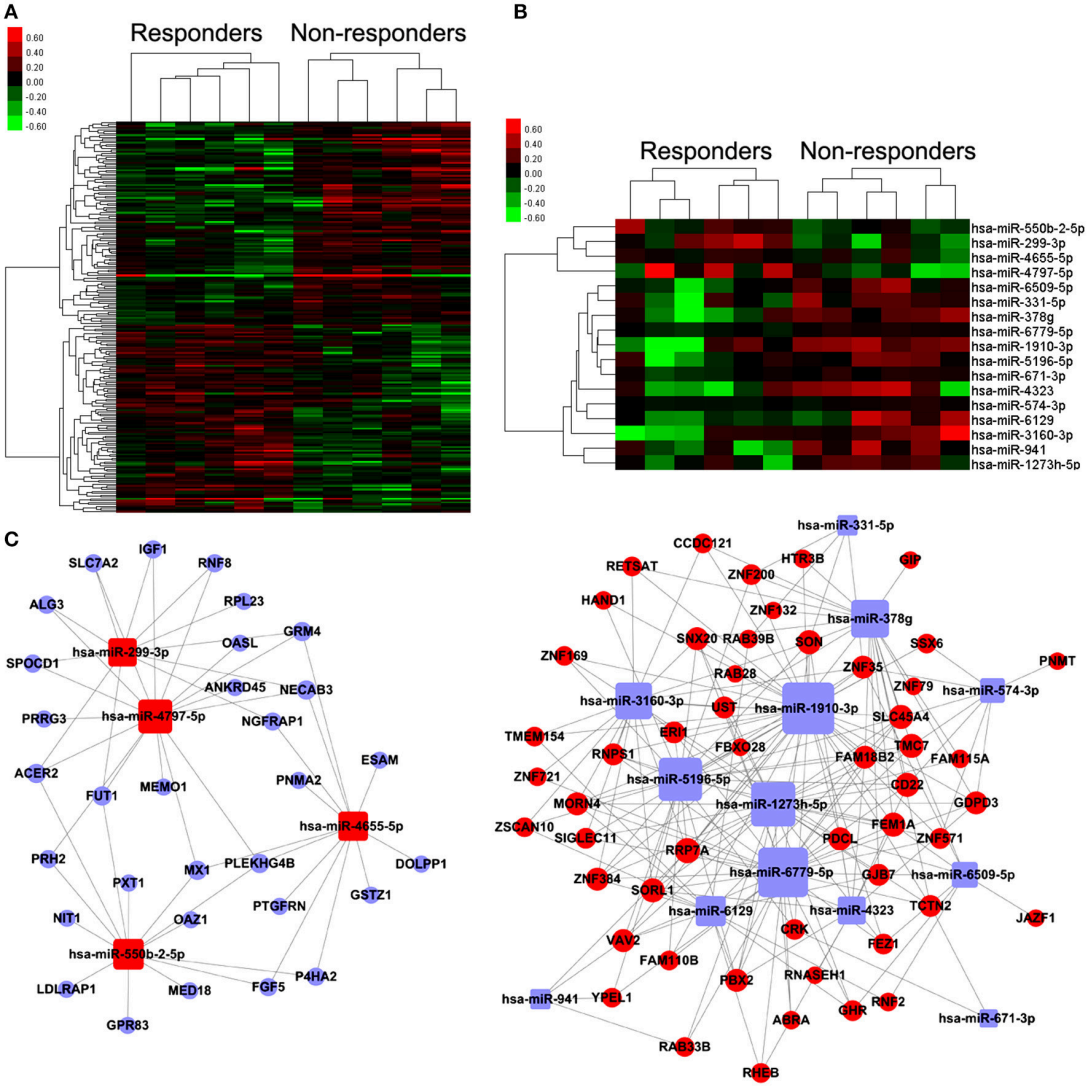
Differentially expressed mRNAs and miRNAs, and miRNA-mRNA coexpression network associated with response to TG tablets. **(A,B)** Heat map showing hierarchical clustering of mRNAs and miRNAs, whose expression changes were more than 1.5-fold in the comparison between the responder and non-responder groups. In clustering analysis, up- and down-regulated genes are colored in red and green, respectively. **(C)** miRNA-target gene co-expression network constructed using the differentially expressed miRNAs and their corresponding putative targets which were also distinctly expressed between the responder and non-responder groups. Red and blue square nodes respectively refer to the upregulated and downregulated miRNAs in the responder group compared to the non-responder group. Red and blue circle nodes respectively refer to the upregulated and downregulated mRNAs in the responder group compared to the non-responder group. The sizes of the nodes were represented by degree that is the contribution of one miRNA to the genes around or the contribution of one gene to the miRNAs around.

The heat-maps (Figures 2A,B) and the unsupervised hierarchical clustering of the expression profiles of the above differentially expressed miRNA and mRNA profiles revealed distinctive patterns for responders and non-responders to TG tablets.

### Candidate circulating miRNA biomarkers predict response to TG tablets based on the discovery cohort

According to the common prediction results obtained from TargetScan and miRanda, 2,097 pairs of miRNA-putative target gene were obtained. Among them, 257 pairs were selected to construct the miRNA-target gene co-expression network using the following criterion: (1) the putative target genes of differentially expressed miRNAs also showed distinctive expression patterns between responders and non-responders to TG tablets; (2) Levels of differentially expressed miRNAs were negatively correlated with that of the corresponding putative target genes. As shown in Figure 2C, the nodes in the miRNA-target gene co-expression network contained 17 differentially expressed miRNAs and 83 putative target genes, and the edges referred to the negatively regulation between miRNAs and the putative target genes. Following the calculation of the nodes' degree, we found that hsa-miR-378g with the biggest degree was the key miRNA in the network, suggesting its important contribution to the downstream target genes around (Table S4).

**Figure 3:**
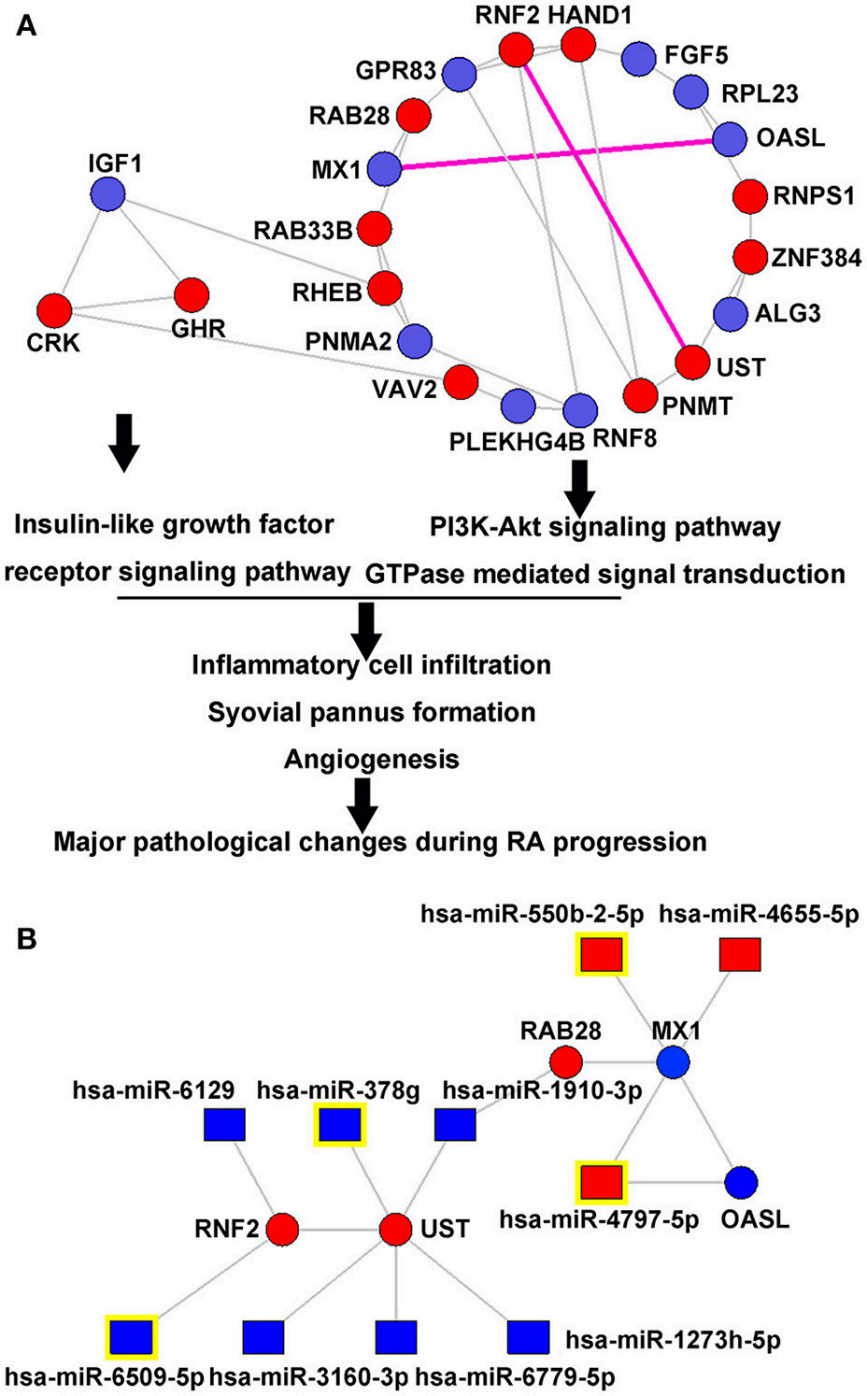
miRNA-mediated gene signal transduction network**. (A)** Two functional modules in the miRNA-mediated gene signal transduction network. The miRNA-mediated gene signal transduction network was divided into two functional modules containing 19 and 3 nodes, respectively. The first module was significantly associated with PI3K-Akt signaling pathway (*P* = 0.02) and GTPase mediated signal transduction (*P* = 0.003), and the second one was involved into Insulin-like growth factor receptor signaling pathway (*P* = 0.02), which can potentially regulate the major pathological changes during RA progression. **(B)** Upstream differentially expressed miRNAs that regulate the interactions of MX1-OASL and RNF2-UST. Red and blue square nodes respectively refer to the upregulated and downregulated miRNAs in the responder group compared to the non-responder group. Red and blue circle nodes respectively refer to the upregulated and downregulated mRNAs in the responder group compared to the non-responder group. Purple edges refer to the gene-gene interaction with the highest edge-betweenness, suggesting their crucial roles in transferring signals. miRNA nodes with yellow marks refer to the most differentially expressed miRNAs that regulate MX1, OASL, RNF2, and UST, respectively.

To characterize drug response by functional organization, the miRNA-mediated gene signal transduction network was constructed using the interaction among 83 putative target genes of differentially expressed miRNAs. Following the calculation of degree, closeness and betweenness centrality values, 11 genes with the three feature values higher than the corresponding median values simultaneously were identified, suggesting their great topological importance in the network (Table S5). After that, the Markov clustering algorithm was used for modularity analysis of the miRNA-mediated gene signal transduction network. Nodes that are highly interconnected within the network are usually involved in the same biological modules. As shown in Figure 3A, the miRNA-mediated gene signal transduction network was divided into two functional modules containing 19 and 3 nodes, respectively. Moreover, the functional enrichment and annotation demonstrated that the first module was significantly associated with PI3K-Akt signaling pathway (*P* = 0.02) and GTPase mediated signal transduction (*P* = 0.003), and the second one was involved in Insulin-like growth factor receptor signaling pathway (*P* = 0.02), which can potentially regulate the major pathological changes during RA progression and have also been indicated as therapeutic targets of this disease (Malemud, 2015; Alunno et al., 2017; Cheung and McInnes, 2017; Figure 3A).

The most important gene-gene interaction was identified by calculating “edge-betweenness” that was defined as a bottleneck which has many “shortest paths” going through it and controls the rate of signal flow. As shown in Table S6, the interactions of MX1-OASL and RNF2-UST had the highest edge-betweenness value, suggesting their importance in transferring signals in the network. Figure 3B illustrated the regulation interaction between the upstream miRNAs, as well as the corresponding putative targets MX1, OASL, RNF2, and UST. According to the microarray data, hsa-miR-550b-2-5p (*Foldchange*_*responders*/*non*−*responders*_ = 1.62, *P* = 0.004), hsa-miR-4797-5p (*Foldchange*_*responders*/*non*−*responders*_ = 1.55, *P* = 0.02), hsa-miR-6509-5p (*Foldchange*_*responders*/*non*−*responders*_ = 0.61, *P* = 0.02) and hsa-miR-378g (*Foldchange*_*responders*/*non*−*responders*_ = 0.45, *P* = 0.001) were the most significantly dysregulated miRNAs targeting MX1, OASL, RNF2, and UST, respectively. According to the database of GeneCards (http://www.genecards.org/, Version 4.5.1), four miRNA-target gene pairs were functionally involved into several signal pathways in immune system and drug metabolism, such as Cytokine Signaling in Immune system, Innate Immune System, Peginterferon alpha-2a/Peginterferon alpha-2b Pathway, Pharmacodynamics, DNA Damage, Chondroitin sulfate/dermatan sulfate metabolism and Glycosaminoglycan metabolism (Table S7).

Considering the results of both the miRNA-target gene co-expression network and the miRNA-mediated gene signal transduction network analyses, as well as the potentials of circulating miRNA as disease biomarkers, four miRNAs (hsa-miR-550b-2-5p, hsa-miR-4797-5p, hsa-miR-6509-5p, and hsa-miR-378g) were selected as candidate biomarkers of response to TG tablets, and their levels would be used to construct the SVM-based model for the prediction of drug response.

### Validation of candidate circulating miRNA biomarkers based on the validation cohort

**Figure 4:**
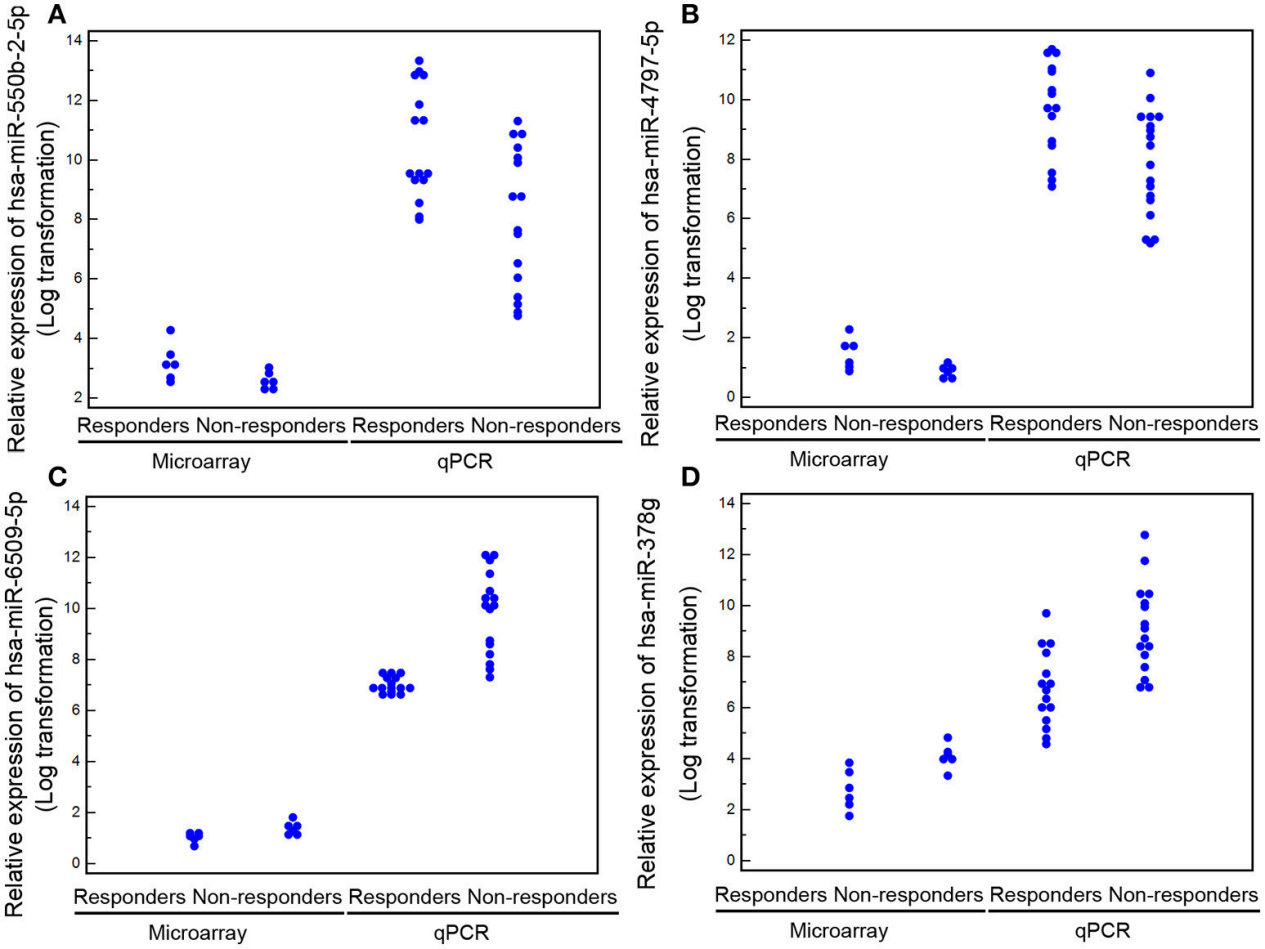
Levels of the four candidate miRNA biomarkers **(A)** for hsa-miR-550b-2-5p; **(B)** for hsa-miR-4797-5p; **(C)** for hsa-miR-6509-5p; **(D)** for hsa-miR-378g) in peripheral blood detected by microarray and quantitative PCR analyses. Data are represented as the mean ± S.D. (*n* = 6 for each group in the discovery cohort; *n* = 16 and 15 for non-responder and responder groups in the validation cohort).

Following the identification of the four most recognized miRNAs as candidate biomarkers of response to TG tablets, we tried to verify the microarray data in the validation cohort of 31 patients by quantitative PCR analysis. Consistently, all the four miRNAs showed clearly distinguished expression in PBMCs obtained from the responder and non-responder groups with high confidence level (*Foldchange*_*responders*/*non*−*responders*_ of hsa-miR-550b-2-5p = 5.25, *P* < 0.001; hsa-miR-4797-5p = 5.70, *P* < 0.001; hsa-miR-6509-5p = 0.25, *P* < 0.001; hsa-miR-378g = 0.23, *P* < 0.001; Figure 4).

### The SVM-based model efficiently predicts response to TG tablets

The SVM-based model for predicting response to TG tablets was constructed based on the levels of the four candidate miRNA biomarkers. The discovery cohort was used to perform 5-fold cross-validation to evaluate the performance of this model. As a result, the accuracy values of the model in the five tests were respectively 83.33%, 100.00%, 100.00%, 83.33% and 100.00%, and the AUC values were all 1.0. In the independent test validation, the expression levels of four candidate miRNA biomarkers (hsa-miR-550b-2-5p, hsa-miR-4797-5p, hsa-miR-6509-5p, and hsa-miR-378g) in peripheral blood of 31 RA samples containing 16 non-responders and 15 responders to TG tablets were used to validate our SVM-based model. As a result, the accuracy and AUC values of the model were respectively 90.32% and 1.000. These data indicated the great reliability and efficacy of this model to screen responders to TG tablets from RA patients against different test datasets.

**Figure 5:**
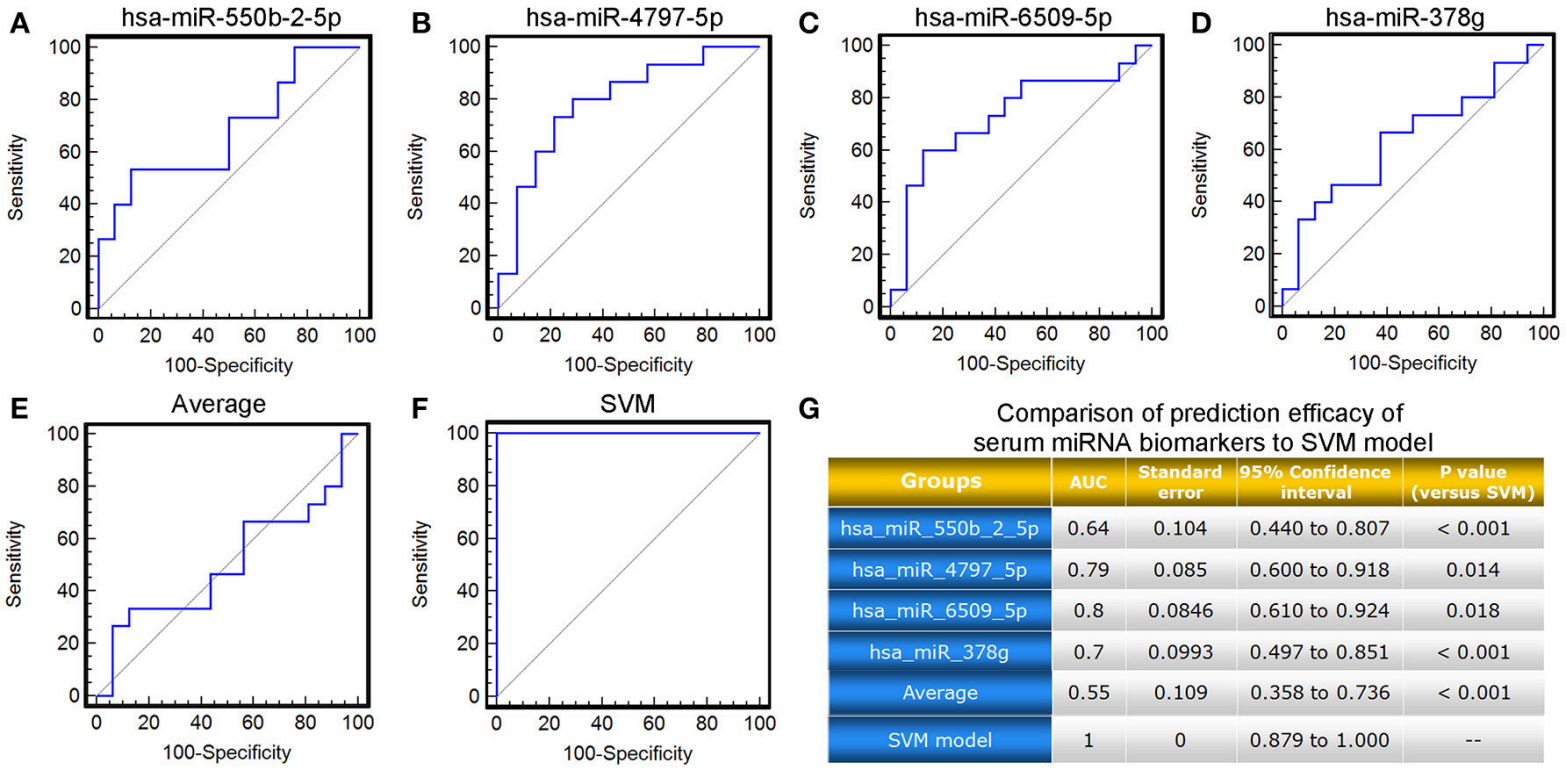
ROC comparison of the four candidate miRNA biomarkers, their average level with the SVM-based model in predicting response to TG tablets**. (A–F)** ROC curves of hsa-miR-550b-2-5p, hsa-miR-4797-5p, hsa-miR-6509-5p, hsa-miR-378g, the average of their levels, the SVM-based model in predicting response to TG tablets. **(G)** Statistical significance in ROC comparisons of the four candidate miRNA biomarkers, their average serum level with the SVM-based model in predicting response to TG tablets.

As mentioned above, the four candidate miRNA biomarkers used in combination for our SVM-based model were selected due to the great contributions to the downstream target genes around in the miRNA-target gene co-expression network, as well as the topological importance and relevance of their target genes in the miRNA-mediated gene signal transduction network. To verify the rationality of this design, we compared the performance of our SVM-based model with the four candidate miRNA biomarkers alone based on the validation cohort. As shown in Figure 5, neither single miRNA nor the average of miRNA levels displayed better power in predicting response to TG tablets than the SVM-based model combining the four candidate miRNA biomarkers (AUC value comparisons, all *P* < 0.05).

**Figure 6:**
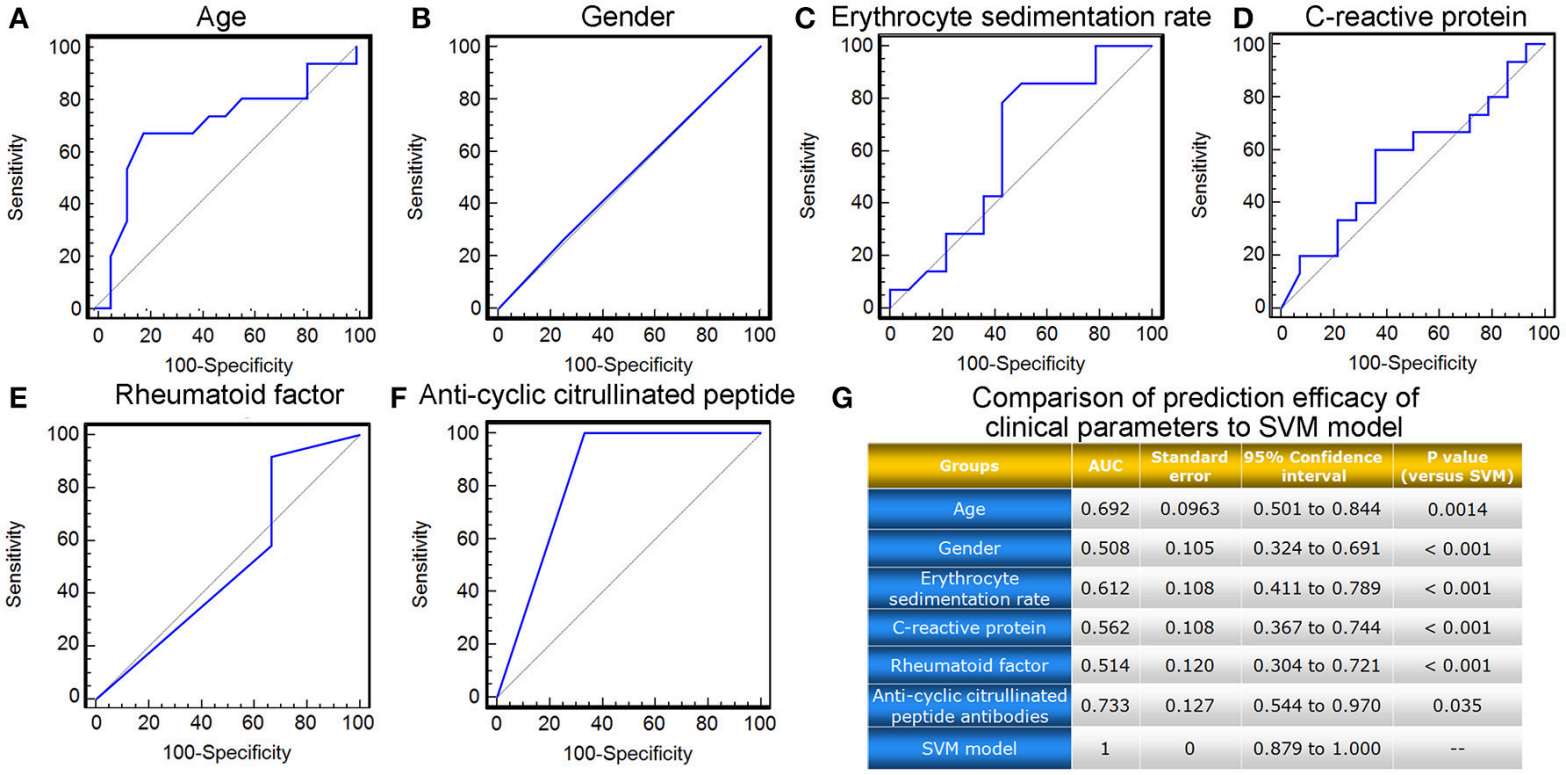
ROC comparison of various clinical parameters with the SVM-based model in predicting response to TG tablets. **(A–F)** ROC curves of age, gender, erythrocyte sedimentation rate, C-reactive protein, rheumatoid factor and anti-cyclic citrullinated peptide antibodies in predicting response to TG tablets. **(G)** Statistical significance in ROC comparisons of various clinical parameters with the SVM-based model in predicting response to TG tablets.

Moreover, we also compared the prediction efficacy of the SVM model with various commonly used clinical and inflammatory parameters, including patients' age, gender, erythrocyte sedimentation rate (ESR), as well as levels of C-reactive protein (CRP), rheumatoid factor (RF), and anti-cyclic citrullinated peptide (CCP) antibodies. ROC comparison analysis demonstrated the marked better performance of the SVM model based on the levels of four candidate miRNA biomarkers in peripheral blood than the clinical and inflammatory parameters (AUC value comparisons, all *P* < 0.05, Figure 6).

These findings demonstrated a distinguished improvement of the SVM-model based on the levels of four candidate miRNA biomarkers in combination over commonly used clinical and inflammatory parameters, as well as miRNA biomarkers alone, in predicting RA patients' response to the treatment of TG tablets, suggesting the necessity and the effectiveness of this model construction.

## Discussion

An increasing number of clinical evidence show that not all RA patients benefit to the same extent from the TwHF-based therapy, suggesting that it is of great significance to develop predictive biomarkers and tools for determination of patients who may have a low probability of response to the TwHF-based therapy, so as to allow clinicians to choose alternative drugs at an earlier stage of the disease without any delay of efficacious treatment, in line with the concept of precision medicine (Bluett and Barton, 2017). Here, we integrated the miRNA and mRNA expression profiles, miRNA target prediction, miRNA-target gene coexpression, as well as gene-gene interactions, to identify four candidate miRNA biomarkers that were predictive of response to TG tablets. Moreover, we applied the SVM algorithm to construct the prediction model for the treatment outcome of TG tablets based on the levels of the candidate miRNA biomarkers in peripheral blood, and also confirmed its good performance via both 5-fold cross-validation and the independent clinical cohort validations. To the best of our knowledge, this is the first study that identified miRNAs in peripheral blood as predictive biomarkers of TG tablets' response in patients with RA and also evaluated their utility in the prediction model of the clinical treatment outcome.

Network biology is a useful strategy to systematically understand disease occurrence and progression, as well as therapies and drug responses. Here, we successfully introduced network approaches to explore the molecular properties of TG tablets' response. On the basis of the miRNA and mRNA expression profiles obtained from responders and non-responders, we uncovered two networks: the miRNA-target gene co-expression network revealed the relationships between miRNAs and the potential target genes counted by their differential expression values, and the key miRNAs were screened according to the degree referring to the contribution of one miRNA to the target genes around; The miRNA-mediated gene signal transduction network depicted the interactions among the differentially expressed target genes of the dysregulated miRNAs, and the topological important target genes were screened. Considering the distinguishing expression patterns and the network-based characteristics, we identified hsa-miR-550b-2-5p, hsa-miR-4797-5p, hsa-miR-6509-5p, and hsa-miR-378g as candidate biomarkers predicting individual response to TG tablets. According to the functional enrichment analysis and literature retrievals, these miRNAs and the corresponding target genes were associated with RA pathogenesis and drug metabolism (as shown in Table S7). The target gene of hsa-miR-550b-2-5p (*MX1*) and the target gene of hsa-miR-4797-5p (*OASL*) both function as interferon (IFN-I)-inducible genes and are predominantly involved into signal pathways in the immune system. The IFN-I signatures in RA may display clinical relevance in relation to disease onset and therapeutic response (Hua et al., 2006; de Jong et al., 2016). Sanayama et al. (2014) identified *MX2* (a member in the same family with MX1), and *OASL* as biomarkers for predicting the therapeutic response to tocilizumab in RA patients. *RNF2* (the RING domain-containing E3 ubiquitin-protein ligases RING finger protein 2), the putative target of hsa-miR-6509-5p, is involved in the maintenance of histone H2A levels and impacts transcriptional activity (Wang et al., 2004). RING E3 ligases were involved into the control of multiple cellular processes and also regarded as candidate therapeutic target of RA (Yagishita et al., 2012). The ubiquitin/proteasome protein degradation pathways were found to offer a contribution to prolonging the survival of synovial fibroblasts in RA tissue (Li et al., 2014). Interestingly, Torre et al. (2017) found that a E3 ubiquitin ligase might positively regulate type I interferon responses and promote pathogenesis during neuroinflammation, implying several possible associations of *RNF2* with *MX1* and *OASL*. The putative target gene of hsa-miR-378g, *UST*, is involved in Chondroitin sulfate/dermatan sulfate metabolism and Glycosaminoglycan metabolism; Chondroitin sulfate on cartilage surface is the long sought high-affinity receptor for glucose-6-phosphate isomerase, the binding of which to the cartilage surface is a prerequisite for autoantibody-induced joint-specific inflammation (Zhou et al., 2010). As one of the extracellular matrix components, changes in glycosaminoglycans have been reported to play a significant role in the RA pathomechanism, and may be related to the disease activity (Jura-Półtorak et al., 2014). The above literature reports support the evidence that the candidate miRNA biomarkers identified in the current study may be associated with disease progression and treatment outcome of RA.

In order to determine the clinical utility of the candidate miRNA biomarkers, we built the prediction model for TG tablets' treatment, using a supervised machine learning algorithm SVM, which can address the general case of non-linear and non-separable classification efficiently (Chen et al., 2011). Since its performance has been indicated to be strongly dependent on the selection of kernel functions, and our preliminary study of the comparison in the SVM models with different kernels (including linear, quadratic, polynomial and radial basis function) demonstrated the best performance of radial basis function, we chose this function in our model. Notably, both the cross-validation and the independent clinical cohort validation suggested that this model may be capable to predict the therapeutic effectiveness in RA patients treated with TG tablets, and also confirmed the essentiality of the model construction by comparing the performance of each miRNA biomarker alone and the model with miRNA combination.

In conclusion, this circulating miRNA-based biomarker model may assist in screening TG tablets' responsive RA patients and thus potentially benefit precision therapy of RA in a daily clinical setting. The weak point of this study is the relatively small sample size for the generation and validation of the predictive model, which may lead to some model over-fitting and thus, potential overestimation of effect size. Thus, future studies based on large clinical cohorts to verify the utility of this model in predicting and monitoring TG tablets' treatment outcome are needed. Moreover, the risk of reversible reproductive toxicity is of great concern when TwHF-based therapy is used for the treatment of RA patients. We also intend to perform a long-term study based on large clinical cohorts to evaluate its safety and identify biomarkers to predict the patients' response to its side effects.

## Author contributions

NL and QJ conceived of the study, and participated in its design and coordination. YZ designed the study, performed the data analysis and drafted the manuscript. HW and GL carried out the clinical sample collection and drafted the part of manuscript. The other authors participated in the clinical sample collection and performed the statistical analysis. All authors read and approved the final manuscript.

### Conflict of interest statement

The authors declare that the research was conducted in the absence of any commercial or financial relationships that could be construed as a potential conflict of interest. The reviewer SM and handling Editor declared their shared affiliation.
