# Supplementary material for: A Novel Circulating miRNA-Based Model Predicts the Response to Tripterysium Glycosides Tablets: Moving Toward Model-Based Precision Medicine in Rheumatoid Arthritis
Source: Front Pharmacol. 2018 May 24;9:378. doi: 10.3389/fphar.2018.00378 (PMC5977984; doi:10.3389/fphar.2018.00378)
Supplement: Supplementary file 2 [file Table_2.DOCX]

**Table S2 Primers of miRNAs used in quantitative PCR analysis**

| **miRNAs** | **Primers** |
| --- | --- |
| hsa-miR-550b-2-5p_F | cgATGTGCCTGAGGGAGTAAGACA |
| hsa-miR-4797-5p_F | cgGACAGAGTGCCACTTACTGAA |
| hsa-miR-6509-5p_F | gcgATTAGGTAGTGGCAGTGGAAC |
| hsa-U6_F | cgAGTGCAGGGTCCGAGGTATTC |
| hsa-miR-378g_F | cgACTGGGCTTGGAGTCAGAAG |
