# Supplementary material for: A Novel Circulating miRNA-Based Model Predicts the Response to Tripterysium Glycosides Tablets: Moving Toward Model-Based Precision Medicine in Rheumatoid Arthritis
Source: Front Pharmacol. 2018 May 24;9:378. doi: 10.3389/fphar.2018.00378 (PMC5977984; doi:10.3389/fphar.2018.00378)
Supplement: Supplementary file 7 [file Table_7.docx]

**Table S7 Candidate miRNA biomarkers and the corresponding target genes associated with RA-related pathways**

| **miRNAs** | **Target genes** | **Pathways** |
| --- | --- | --- |
| hsa-miR-550b-2-5p | MX1 | Cytokine Signaling in Immune system |
|  |  | Innate Immune System |
|  |  | Peginterferon alpha-2a/Peginterferon alpha-2b Pathway (Hepatocyte), Pharmacodynamics |
| hsa-miR-4797-5p | OASL | Cytokine Signaling in Immune system |
|  |  | Innate Immune System |
|  |  | Immune response IFN alpha/beta signaling pathway |
|  |  | Interferon Signaling |
| hsa-miR-6509-5p | RNF2 | Cellular Senescence |
|  |  | SUMOylation of DNA damage response and repair proteins |
|  |  | Metabolism of proteins |
|  |  | Chromatin Regulation / Acetylation |
|  |  | DNA Damage |
| hsa-miR-378g | UST | Chondroitin sulfate/dermatan sulfate metabolism |
|  |  | Glycosaminoglycan metabolism |
|  |  | Metabolism |
